# Progress in Obstetrics

**Published:** 1899-06-10

**Authors:** 


### Progress in Obstetrics.

(*Continued from page* 167.)

**The Treatment of Puerperal Fever.** — Christopher Martin,[4] of Birmingham, writing on this subject, says he includes under the term puerperal fever the numerous inflammatory lesions in the pelvis, with varying degrees of blood poisoning occurring during the puerperium, and due to the infection of the genital canal by certain micro-organisms. On being called to a case of puerperal fever the surgeon's first duty is to make a careful bi-manual examination of the pelvis, in order to ascertain, if possible, the extent and severity of the local inflammation. The lesions in the abdomen and pelvis which he must look out for are the following: (1) Endometritis and metritis; (2) salpingitis, pyosalpinx, and ovarian abscess; (3) pelvic cellulitis and abscess of the broad ligament; and (4) peritonitis, localised in the pelvis, or general. He considers first the treatment of these local pelvic lesions.

I. *Endometritis.*—This, which he believes is the commonest lesion occurring in puerperal fever, is mainly characterised by (1) a swollen and tender uterus; (2) offensive lochial discharge; and (3) symptoms of sapræmia or septicæmia. He advocates curetting the uterus, irrigating with iodine or some other antiseptic solution, and the application of iodised phenol to the interior of the uterus.

II. *Salpingitis, Pyosalpinx, and Pelvic Peritonitis.*—In these cases the infection has spread into the Fallopian tubes, setting up salpingitis or pyosalpinx, and through the tubes into the pelvic peritoneum, setting up a localised pelvic peritonitis. In such cases, if there be evidence of a collection of fluid (sero-pus or pus) either behind the uterus (in the pouch of Douglas) or at either side of the uterus (in the Fallopian tubes), he recommends posterior colpotomy, making a free incision and draining. He does not advocate the more severe operations recommended by some American and Continental surgeons of vaginal extirpation of the uterus and Fallopian tubes, and points out that these patients are not in a condition to stand a severe operation, and there is great danger of converting a localised infection into a general one by pumping a large amount of toxic material either into the peritoneal cavity or into the general circulation.

III. *Pelvic Cellulitis.*—In some cases it will be found that the cellular tissue of one or both broad ligaments is infected and inflamed. On one or both sides of the uterus low down in the pelvis, and extending out to the lateral pelvic wall, a hard, broad-like mass of infiltration is felt, fixing the uterus as in a mass of plaster of Paris. If this does not go on to suppuration he recommends hot vaginal douches every six hours, and after each douche a tampon soaked in ichthyol and glycerine (10 per cent.) should be introduced into the upper part of the vagina. As soon, however, as an abscess forms it should be opened.

IV. *General Peritonitis.*—In these cases there is a hard, drummy distension of the abdomen, constipation, vomiting, a pinched face, dry tongue, and quick pulse. He recommends the administration of calomel by the mouth, and repeated turpentine enemata. Five grains of calomel should be given every six or eight hours until the bowels move freely. If there be signs of free fluid in the peritoneum the question of opening the abdomen has to be considered. If on vaginal examination fluid can be felt bulging behind the uterus and distending the pouch of Douglas, the peritoneum should be opened through the posterior fornix, and an iodoform gauze or rubber drain inserted. In several cases Martin has opened the abdomen above the pubes, and washed out and drained the peritoneum. Turning now to the general treatment of puerperal septicæmia, he says we have (1) to treat urgent symptoms as they arise, (2) to try and neutralise or eliminate the poison circulating in the patient's blood, and (3) to maintain the patient's strength during the process. For the treatment of high fever nothing answers so well as quinine; if the patient be vomiting it should be administered as an enema, or, better still, by hypodermic injection. If she can retain it, the drug may be given by the mouth in five-grain doses every six, eight, or twelve hours, according to the severity of the fever. Quinine not only reduces the temperature, but in some way acts as an antitoxin in the blood. In cases of hyperpyrexia he recommends cold sponging. Heart failure should be combated by the administration of digitalis and strychnine, either by the mouth or hypodermically. Alcohol must be administered freely. If the patient can retain it, brandy and milk or egg-flip should be given by the mouth. If she be vomiting, brandy and beef-tea enemata should be administered every four hours. If there be diarrhœa no attempt should be made to check it, as the poison is in this way eliminated. Should there be no diarrhœa the bowels should be opened with calomel (five grains), followed by a saline aperient, such as sulphate of magnesia.

Within recent years a new method of combating the general blood poisoning has been introduced in the form of antistreptococcic serum. The serum is injected into the subcutaneous tissue of the abdominal wall. In ordinary cases one dose of 10 c.c. is administered every twenty-four hours, in severe cases it is advisable to administer it every twelve hours. Antistreptococcic serum is efficacious only in those cases of puerperal fever in which the streptococcus pyogenes is the predominant infecting agent. For example, in cases of puerperal fever due to the influence of staphylococci, gonococci, the bacillus coli communis, or the bacillus of diphtheria it is not likely that the antistreptococcic serum will prove of much value; but most cases of puerperal fever are cases of mixed infection, and in the majority of these the streptococcus pyogenes is the predominant germ. Lastly, he thinks the injection of saline solution in large quantities into the subcutaneous tissue or the deep cellular tissue under the mamma is undoubtedly of value; from a pint to a quart of normal saline solution is slowly injected into the cellular tissue, and this may be repeated every twelve hours. Being quickly absorbed into the blood it dilutes it, and so helps it to eliminate through the skin, kidneys, and intestina tract any toxin it may contain. With regard to the treatment by antistreptococcic serum, Dr. Eden[5] emphasised the importance of restricting the use of antistreptococcic serum to cases of this particular infection, and referred to Haultain's paper relating to three cases of septicæmia. In one case Loeffler's bacillus was found, which was treated with antidiphtheritic serum and recovered. In the second case the streptococcus was found, and the patient recovered under the antistreptococcic serum treatment; but in the third case the bacillus coli communis was present, and as there was no special serum the patient died. Cullingworth[6] urges that the antistreptococcic serum ought to be used in every case of septicæmia, though he admits that many cases are due to mixed infection. If, he says, we are not fully equipped to fight this disease, that is not *per se* a reason for not making use of such arms as we have at our disposal. Dr. McCann[7] suggests that the serum should be used as a preventive measure where there is reason to apprehend subsequent infection, as, for instance, in the case of parturient patients admitted with a foul discharge. Saunders[8] reports five cases of streptococcic infection in which he used the serum, and says that it has no antitoxic properties, but probably stimulates phagocytosis, and possibly renders the plasma capable of more or less paralyzing the micrococci. He attributes some of the poor results observed in the past to the following causes: (1) The use of worthless or inferior serum; (2) the existence of mixed infection; (3) delay in commencing the treatment. He says that an absolutely fresh serum is needed—not over two or three weeks old—as these serums rapidly deteriorate.

**Cæsarian Section.**—Robert Jardine,[2] in a paper on this subject, after reading notes of four of his cases, refers to the following points in the operation: (1) How are we to control uterine hæmorrhage during incision? He says an elastic ligature, in his opinion, does more harm than good. Cameron's method of pressing a slightly curved pessary firmly on the uterus and cutting inside of it does admirably. (2) How should the uterine incision be made? A transverse fundal incision has been recommended, and also a longitudinal one right over the fundus; but he thinks a longitudinal one, beginning at the fundus and extending downwards for about four inches, is better than either of these. (3) Which is the best material to suture with? In his cases he used silk, and says the ease with which it can be rendered aseptic by boiling is greatly in its favour; but he points out that sometimes the sutures suppurate out months after the operation, and he suggests catgut, which he thinks would last quite long enough. Dr. Braithwaite[3] also recommends green chromic catgut, soaked for half an hour in perchloride solution, 1-1,000, as the best material for suture of the uterine wall.

**Puerperal Eclampsia.**—Robert Jardine[9] recommends intercellular saline injections in the treatment of puerperal eclampsia, the aim being to flush the kidneys and thus rid the system of the poison, whatever it may be, which gives rise to the convulsions. The diuretic action, he says, has been very marked in all the cases which recovered, or lived long enough to give it time to act. The apparatus required is a very simple one, viz., a medium-sized trocar and cannula, six feet of small tubing, a glass filter, and a small piece of adhesive plaster. An ordinary trocar and cannula will do, but one from an aspirator does much better. The saline solution is made by adding a teaspoonful of equal parts of common salt and bicarbonate of soda to a pint of boiled water; the temperature should be 100 deg. Fah., and he injects it into the axilla or under the breasts, but it may be done into any part of the body where the tissues are lax; strict aseptic precautions should be taken. A large swelling usually forms, and there is considerable tension in it; if it is in the axilla the patient will complain of pain and numbness in the arm from pressure on the nerves, but this soon passes off. A pint of solution will be absorbed in from ten to fifteen minutes. In a number of cases direct transfusion into a vein was adopted, but Dr. Jardine is not satisfied that results in these cases have been so good; moreover, the intercellular method is much easier, requires no cutting, there is no wound to deal with afterwards, and there is no risk of introducing air into the circulation. The absorption takes place through the lymphatic system, and by the time the fluid reaches the circulation it will be in a better condition to mix with the blood than when thrown directly into the vessel.

[2] Scottish Med. and Sur. Jour., Nov., 1898, Robert Jardine. [3] Lancet, Nov. 5, 1898, Dr. Braithwaite. [4] Birm. Med. Rev., Jan., 1899, Christopher Martin. [5] Med. Press, Oct., 1898, Dr. Eden. [6] Med. Press, Oct. 12, 1898, Cullingworth. [7] Lancet, Oct. 15, 1898, McCann. [8] Amer. Jour. of Obstetrics, Jan., 1899, Saunders. [9] Glasgow Med. Jour., Mar., 1899, Robert Jardine.

